# Therapeutic Approaches and Role of ncRNAs in Cardiovascular Disorders and Insulin Resistance

**DOI:** 10.1155/2017/4078346

**Published:** 2017-09-19

**Authors:** Kalupahana Irushi Pamodya Liyanage, Gamage Upeksha Ganegoda

**Affiliations:** Faculty of Information Technology, University of Moratuwa, Katubedda, Moratuwa, Sri Lanka

## Abstract

Diseases resulting from alterations in gene expressions through mutations in the genes or through changes in the gene expression regulation could be identified through the analysis of RNA expressions. ncRNAs play a significant role in regulation of the gene expression by controlling the expression levels of the coding RNAs and other cellular processes. Discoveries have shown that the human genome is encoded with sequences responsible for the transcription of thousands of ncRNAs. Even though the studies conducted on ncRNAs are still at initial stages, facts established so far display biomarkers that confirm their relationship with certain diseases such as cancers, cardiovascular diseases, and insulin resistance. These studies have been facilitated with high throughput modern sequencing techniques such as microarrays and RNA sequencing. The data obtained through the above analysis are processed with the aid of existing databases, to deduce conclusions on different diagnostic biomarkers and therapeutic targets for specific diseases. This review focuses on the association of ncRNAs in disease prediction, focusing mainly on cardiovascular diseases and disorders caused by insulin resistance. The report also analyzes regulatory functions of ncRNAs and novel approaches used in disease therapeutics.

## 1. Introduction

With the advancement of science and the field of bioinformatics, it is being discovered that many complex diseases ranging from cardiovascular diseases to rare disorders constitute a genetic element, thus, drawing the attention of researchers onto carrying out studies regarding the genetic variations contributing to diseases [1]. This task has been heavily aided by the wide range of biological databases and the advent of high throughput genomic tools and technologies. Analysis of multiple molecular targets such as genes, proteins, coding, and noncoding RNAs is employed in complex disease predictions [2]. This review focuses on the role of noncoding RNAs (ncRNAs) in the regulation of complex processes leading to cardiovascular diseases and pathological complications caused by insulin resistance, current findings, and different technologies used in this emerging area of study.

Until recently, the function of a RNA was assumed to be largely in the form of mRNAs, tRNAs, and rRNAs [3]. However, with the advent of research it has been revealed that humans and mice have roughly the same amount of protein coding genes as a microscopic round worm, leading to the question as to how the biological complexity of organisms is sequenced in the genome [4]. The answer to this question is that the biological complexity is correlated with the amount of noncoding genes. It has also been discovered that not more than 2% of the mammalian genome is transcribed in the form of protein coding RNAs, whereas the rest is in the form of non-protein-coding RNAs [5]. Thus, leading to the realization of how important the function played by ncRNAs could be.

Unlike sequences of protein coding genes, various types of noncoding RNAs, which have been measured with low importance in terms of functional relevancy until the recent past, have shown significant contribution in the regulation of various molecular processes, under the modern findings [6]. This has elevated the need for studies that could discover relationships among ncRNAs and complex diseases such as tumorigenesis, cardiovascular disorders, neurological disorders, and diabetes [7].

Among the vast number of ncRNA types microRNAs (miRNAs), small nucleolar RNAs (snoRNAs), PIWI interacting RNAs (piRNAs), large intergenic noncoding RNAs (lincRNAs), and transcribed ultraconserved regions (T-UCRs) are emerging as key components of cellular homeostasis [2, 8]. Gene regulation, germ cell reprograming, transcriptional and posttranscriptional gene slicing, paramutation, pathogenesis of diseases, and X chromosome activation in mammals are some of the identified complex molecular activities carried out by different ncRNAs [5]. Epigenetic and genetic events which can cause disruption of ncRNAs and its related proteins have shown the possibility of creating pathological outcomes [9, 10]. Currently, research studies are being carried out in order to establish the relationships of these ncRNAs with diseases and to discover therapeutic approaches to offset the effect of disease causing ncRNAs.

This review will include, in detail, an explanation of the role of ncRNAs in pathology, insulin resistance, and cardiovascular diseases, as well as the linkage of cardiovascular diseases and insulin resistance and therapeutic approaches available in the current world. A discussion and a conclusion on this review are included in Sections 9 and 10, respectively.

## 2. Role of ncRNAs in Pathology

Noncoding RNAs are broadly categorized into structural and regulatory, based on the role they play in cellular processes. Structural ncRNAs include ribosomal RNAs (rRNAs) and transfer RNAs (tRNAs) whereas regulatory RNAs could be further divided into three categories based on their length, namely, small, medium, and long. Small ncRNAs include ncRNAs such as microRNAs (miRNAs), PIWI interacting RNAs (piRNAs), and small interfering RNAs (siRNAs). Medium ncRNAs include ncRNAs such as small nucleolar RNAs (snoRNAs) and tiRNAs [9]. Long ncRNAs include ncRNAs such as lincRNAs, eRNA, PALRs, and NATs [9]. These could be further classified into different classes depending on their functionality, length, and the three-dimensional folding. The attention which has been on small ncRNAs such as ribosomal RNA (rRNAs) and transfer RNAs (tRNAs) over the past years has now changed to small ncRNAs such as microRNAs (miRNAs), small nuclear RNAs (snRNAs), and circular RNAs (circRNAs), which are involved in the regulation of cellular activities in eukaryotes. Described below in this review are some of the well-studied ncRNAs that are assumed to be playing a role in the pathological processes. It is identified that there exist millions of distinct piRNA sequences, more than 700 miRNAs, and hundreds of siRNAs [5]. However, the true potential of ncRNAs in the regulatory activities is yet to be discovered.

### 2.1. miRNAs

miRNA expressions are highly conserved, tissue specific, and tightly controlled during embryogenesis [9, 10]. One of the main functionalities of these small endogenous ncRNAs includes silencing of mRNAs through translational repression or through transcript degradation. They are of around 22 nucleotides in length and their over/underexpression could cause or reflect pathological conditions in the respective tissues, leading to their usefulness as biomarkers for diseases. miRNAs play a regulating role in cell proliferation, development, differentiation, metabolism, apoptosis, aging, and pathophysiology of diseases [10]. Both* in vitro *and* in vivo* modulation of miRNA expressions have revealed their role in the regulation of heart function, cardiac growth, hypertrophy, and heart failure [10]. miRNAs are also found to be playing a major role in the pancreatic *β*-cells, not only during the regulation of insulin secretion but also in the proliferation of *β*-cells [11].

### 2.2. lncRNAs

Identification of lncRNAs are still in the research stage and they are presently categorized as all noncoding RNAs longer than 200 nucleotides [12]. Since most of their functionalities are yet to be discovered, they are assumed to consist of numerous and functionally different transcripts. Some of these lncRNAs are tissue specific and they contribute to the regulation of gene expression through alternative splicing, epigenetic modifications of the DNA, posttranscriptional gene regulation, mRNA stability, and translation [13]. With the research conducted so far, it has been identified that their functionalities include acting as blockers of transcription, scaffolds for chromatin modifiers, microRNA sponges, and antisense RNA [14]. Even though most of their characteristics are unknown, the studied areas have shown relationships with cardiovascular diseases and their possible effects on insulin resistance as described in the later sections of this review.

### 2.3. circRNAs

circRNAs are a type of competing endogenous RNAs (ceRNAs) that can act as miRNA sponges. Their functionality as miRNA sponges indirectly makes them regulators of the protein coding gene expressions allowing them to be used as diagnostic or prognostic biomarkers of diseases and have potential therapeutic values. These stable regulatory RNAs, which are abundantly expressed (10 times that of corresponding linear miRNAs), are predominantly found in the cytoplasm [15]. They are evolutionarily conserved sequences and are mainly derived from the exons of protein coding genes. Their covalently closed looped structure minimizes the degradation caused by RNase R [15]. circRNAs too are tissue specific or development stage specific similar to miRNAs. circRNAs are capable of getting miRNAs bind to it, resulting in the reduction of miRNA activity in the cell. A single circRNA contains many binding sites for miRNAs. Some major types of circular RNAs identified so far include ciRNAs, circRNAs, and ElciRNAs. circRNAs regulate the parental gene expression whereas EIciRNAs and ciRNAs promote the transcription of parental genes. ciRNAs have fewer binding sites for miRNAs [15].

## 3. Insulin Resistance

Insulin resistance is the biological condition where normal or elevated levels of insulin do not show the expected response in the blood glucose level or in other words the reduction in sensitivity of an organism's cells to insulin levels [16, 17]. Insulin is a peptide hormone that facilitates the cellular glucose uptake and maintains the blood glucose level, which in turn helps in regulating the metabolism of carbohydrates, proteins, and lipids in the body [16]. Insulin is created in *β*-cells of the pancreatic islets of Langerhans and the study of diseases caused by complications in insulin resistance remains to be at the forefront of medicine [18]. Insulin resistance could lead to diabetes mellitus which is a chronic metabolic disease making people vulnerable for many other diseases [19]. Insulin resistance leads to elevated blood glucose levels and the cellular activities are impaired due to the lack of energy that could be obtained from glucose. Long term effects of diabetes mellitus or even insulin resistance have shown mortality in patients through diseases such as cardiovascular diseases [17, 19].

Insulin resistance shows clinical syndromes such as type 2 diabetes, hypertension, cardiovascular diseases, nonalcoholic fatty liver, polycystic ovary syndrome, and sleep apnea [16]. Currently the most common clinical method used for the identification of insulin resistance is measuring of the plasma insulin level under fasting conditions [16]. However, the seriousness of implications caused by insulin resistance to the development of other related diseases such as cardiovascular diseases has led to experiments on better therapeutic approaches.

## 4. Role of ncRNAs in Insulin Resistance

ncRNAs which are regulators of cellular processes have been discovered to modulate certain pathological and physiological pathways in diseases such as cardiovascular diseases, diabetes, and cancers [17]. Research conducted with the intention of observing relationships with ncRNAs and insulin resistance has revealed certain relationships such as miRNAs in the *β*-cell development and its functions, insulin production, insulin secretion, and glucose homeostasis [17, 20]. This section of the review focuses on the underlying causes affecting the insulin resistance while giving prominence to how it could be related to ncRNAs.

The importance of ncRNAs on the field of pathogeny lies in their contribution towards diagnostic characteristics of different diseases, which could be either used to understand the underlying cause of the disease or used in the development of therapeutic approaches. The significant effect of miRNAs on insulin resistance is evident through novel experimental results [21]. For instance, the discovery of miR-375 has led to the understanding of its role in glucose homeostasis and maintenance of the pancreatic *β*-cell mass [20]. Furthermore, lncRNAs have not gained noteworthy attention with respect to insulin resistance (compared to miRNAs); however it should be noted that they show a high level of tissue specificity and are known to function in both* cis* and* trans* nature [14].

Table 1 summarizes some of the experimental findings related to ncRNAs affecting insulin resistance. It should be noted that there are more miRNAs that have been identified to show relationships with insulin resistance and that this review aims to give an overview of them.

## 5. Cardiovascular Diseases

Cardiovascular diseases, being the most prominent cause of adult morbidity and mortality worldwide, according to the world health organization, have been gaining a lot of attention in the field of bioinformatics to uncover better prognostic, diagnostic, and therapeutic tools. Given an understanding on the functionality of regulatory ncRNAs, it can be speculated that the analysis of these small noncoding RNAs could lead to a better understanding of the cellular mechanisms related to the disease and identification of more advanced biomarkers.

Some of the major cardiovascular diseases include myocardial infarctions, heart failures, atherosclerosis, restenosis, cardiac senescence, myocardial ischemia, and coronary artery disease [10, 24, 30]. Cardiovascular diseases could be broadly categorized into two, namely, vascular disorders and cardiac disorders, both of which appear to be influenced significantly by changes in the expression levels of regulatory RNAs [13].

Even though there has been a lot of research conducted in this area, which has led to the conclusion that ncRNAs in fact do show a relationship with cardiovascular diseases, there happens to be a wide range of ncRNAs whose functionalities are yet to be discovered [13]. This section focuses on giving an overview of experiments conducted in this research area and a summary of major ncRNA biomarkers that could be used in cardiovascular physiology.

### 5.1. Heart Diseases

The formation of the heart initiates with fusion of myocardial and endocardial cell layers to form a bilayered heart tube that contracts spontaneously to provide blood supply to the developing embryo, which then develops into a complex structure with four chambers [13]. As for most other organs of the body, the development of this first organ during vertebrate embryogenesis is tightly regulated with use of miRNAs and other ncRNAs, during the posttranscriptional stages of the gene transcription [30].

Hata, in her study, has conducted different experiments using mice, to analyze the importance of genes encoding different enzymes essential for the biogenesis of miRNAs such as Dicer, Drosha, DGCR8, and Ago2 [13]. It has been revealed through her research that mice subjected to mutations in key miRNA processing genes have died with critical developmental defects in the heart [13]. The reference articles [9, 13] provide elaborations on experiments conducted and the relationships deduced through them. The severity of the effects shown by mutated genes in mice [13] leads to the prediction that even slight changes in expression of ncRNAs could have major impacts on the correct development and functioning of the heart.

Even though experiments at present are more focused towards miRNAs, it should be noted that other ncRNAs, such as circRNAs and lncRNAs that have the ability to function as miRNA sponges, do have a direct impact on the regulating functions of miRNAs [2, 5]. The ncRNAs discovered so far have shown to contribute largely towards cellular regulatory functions, leading to the possible assumption that ncRNAs that are yet to be discovered too could show similar properties [2, 5, 6].

### 5.2. Vascular Diseases

The vascular system which comprises endothelial cells (EC) and vascular smooth muscle cells (VSMC), forming into a vascular network comprising arteries and veins, seems to be less sensitive towards the regulation of miRNAs as compared to other tissue types [13].

The experiment elaborated in [13] shows that the deletion of gene responsible for endothelial cell specific dicer in mice does not reflect major changes or abnormalities in the angiogenesis of the embryo, leading to the conclusion that miRNAs do not influence the cellular processes in angiogenesis as that of the heart.

However, phenotypic changes such as vascular injuries and stent implantations leading to postnatal angiogenesis have exhibited endothelial cell and vascular smooth muscle cell regulation by miRNAs, leading to chronic inflammatory diseases such as atherosclerosis and restenosis [9].

## 6. Role of ncRNAs in Cardiovascular Diseases

ncRNAs responsible for prognostic, diagnostic, and therapeutic activities are utilized to understand a disease better and develop therapeutic approaches. As discussed before, many ncRNAs are linked with cardiovascular disease conditions. Even though majority of the finding are still on miRNAs, with future experimental revelations on other ncRNAs, promising advances in medicine could be made [31].

Table 2 gives an overview on how some of the recognized ncRNAs affect heart and vascular disorders.

## 7. Linkage of Cardiovascular Diseases and Insulin Resistance

The genome wide studies done on the molecular level activities of cellular mechanisms have enabled the expansion of knowledge on genetic components in pathological diseases, hence improving the scope for development of therapeutic approaches. Over the last few years, diabetes has been recognized as a global epidemic and cardiovascular diseases as the most prominent cause of mortality [32].

Diabetes mellitus is not merely a stand-alone disease but an association of a number of complex diseases. It comes with various microvascular (nephropathy, retinopathy, neuropathy, etc.) as well as macrovascular (coronary heart disease, peripheral vascular disease, stroke, heart failure, etc.) complications [32].

Furthermore, experimental evidences such as miR-208a-MED13 axis showing a potential linkage between metabolic disorders such as type 2 diabetes, obesity, and cardiovascular disease can be seen [13]. According to Ding et al. most patients with cardiovascular diseases have often shown abnormal glucose metabolism towards a relationship between the two diseases [17].

Atherosclerosis is a chronic inflammatory disease of the arteries caused by endothelial cell injury, VSMC proliferation, platelet adhesion, and macrophage and lipid accumulation [17]. Experimental data reveals that atherosclerosis is induced by the abnormal expression of miRNAs resulting due to hyperglycemia [17]. Moreover, cardiovascular diseases such as arrhythmia have been found to be affected by alteration of miRNA levels due to high glucose conditions [12, 17].

Table 3 summarizes some of the instances where relationships are shown between the two disease types.

The factors mentioned in Table 3 show a clear linkage between cardiovascular diseases and insulin resistance. Analysis of this relationship is important before developing novel therapies since individual diseases could be affecting one another and adverse effects could be generated.

## 8. Therapeutic Approaches

Misexpression and mutations in genes, altering the expression of ncRNAs, have shown to be involved in many disease conditions as discussed in the review; hence manipulation of ncRNAs is being experimented as a therapeutic approach in the field of pathology. Gene therapy, ncRNA antagonism, and ncRNA mimics are the most established therapeutic approaches developed in controlling the impacts of ncRNAs [33].

Antisense oligonucleotides and expressed miRNA sponges are used to antagonize the effect of mature miRNAs through degradation or sequestration [33]. This methodology is the most advanced miRNA based therapeutic approach developed so far and uses anti-miRNA oligonucleotides (AMOs). These single stranded AMOs consist of the complementary sequence as that of the target miRNA, thereby disrupting its binding with the respective mRNA and inhibiting the miRNA-mRNA interactions [20]. Different conventional antisense methods could be used to nullify the effects of lncRNAs by silencing them.

In instances where a disease condition is caused due to a deficiency in the amount of expressed miRNAs, then miRNA mimics, which are synthetically developed miRNAs, are employed as a therapeutic approach [33].

Classical gene therapy too is employed as a therapeutic approach to minimize the disease causing effects of ncRNAs.

## 9. Discussion

As per the evidence provided in studies, it is clear that a single ncRNA has the ability to influence many cellular activities. For instance, a single miRNA sponge is capable of affecting multiple mRNAs and sometimes multiple gene sequences could be regulated using a single ncRNA [9]. Therefore, development of novel therapeutic approaches should be done cautiously and only after the entire human transcriptome is revealed. Approaches such as usage of miRNA mimics should be done very carefully since they could be regulating multiple gene sequences.

**Figure 1:**
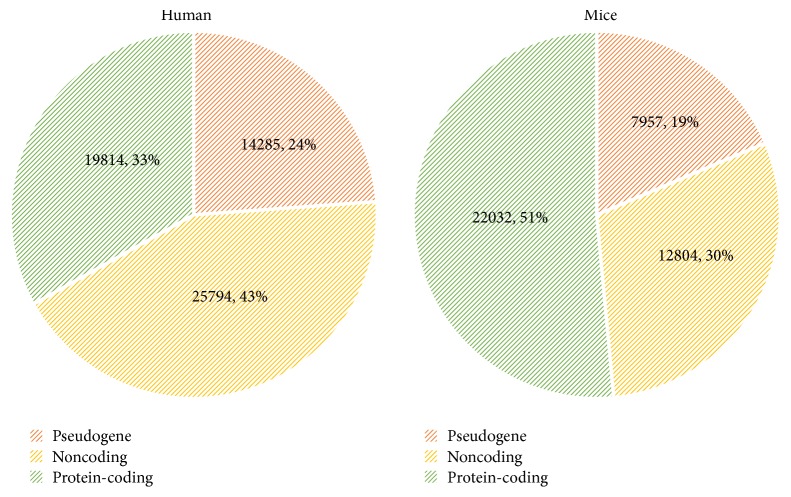
Comparison of human and mice transcriptomes [4].

The advancement of high throughput sequencing technologies has enabled the identification and analysis of ncRNAs, unlike in the past where the attention was predominantly given to protein coding gene sequences. The focus that has been on the protein coding regions of the genome has now therefore shifted to the transcribed noncoding regions of the genome. As the complexity of the mammalian increases, that is, from a less complex being such as a mouse to a complicated being such as a human, the amount of protein coding genes transcribed reduces and the amount of noncoding genes transcribed increases [4]. Leading to the assumption that complexity of beings is regulated mainly through the non-protein-coding gene sequences. The diagram illustrated in Figure 1 clearly portrays the above-mentioned point by presenting the different transcript levels in humans and mice, as per the statistics stated by Kashi et al. [4].

Unlike in the analysis of mRNAs, which are generally longer than 500 nucleotides, analyzing ncRNAs requires sensitive technologies [34]. Also since most of the ncRNAs are not yet well established; the analysis methods should accommodate the identification of novel ncRNAs without the need for a priori information [35]. Although the overall expression of ncRNA transcripts is greater in number than that of mRNAs in a eukaryotic cell [4], there could still be less abundant types of ncRNAs; therefore methodologies should be both high throughput sequencing and sensitive at the same time.

Methods such as real time PCR which has been used mainly for the analysis of miRNA expression levels could also be used for the detection of lncRNAs; however they do not allow the discovery of new ncRNAs [9]. Similarly, RNA immunoprecipitation method, which is used to identify ncRNAs interacting with a specific protein, is unable to make new discoveries. RNA-sequencing is a high throughput sequencing method which can be used to sequence the entire transcriptome, leading to the identification of new ncRNAs [36]. RNA-sequencing has a very high sensitivity in detecting less abundant transcripts and can reveal alternatively spliced isoforms [9, 37]. Microarray analysis is also a high throughput sequencing modern technology and it has a high level of accuracy [38].

However, microarrays are gaining preference over RNA-sequencing due to its cost effectiveness. Genomic tiling arrays are a type of microarray analysis which facilitates the identification of novel ncRNAs in a selected DNA region without a priori knowledge on their precise loci unlike in traditional microarrays [9]. Genomic tiling arrays tend to be particularly useful in the study of lncRNAs.  Table 4 shows a summary of different features present in different types of technologies available for ncRNA analysis.

Insulin maintains the blood glucose level by simulating lipogenesis in adipose tissue, where blood glucose is converted into fatty acids in adipocytes. However, in the case of obesity, macrophage infiltration and inflammatory cytokine release are seen in adipose tissue, which interferes with insulin signaling and inhibits adipogenesis while increasing insulin resistance in peripheral tissues [25, 27]. Studies have shown a relationship between chronic inflammation and the level of miRNAs in adipocytes [27].

Dinarello, in his review, refers to cytokines as regulators of host responses to infection, immune responses, inflammation, and trauma, which can be categorized broadly into proinflammatory (cytokines that act to make diseases worse) and anti-inflammatory (cytokines that promote healing) [39]. The reviews conducted by McGregor et al. have included details on miRNAs and their directly associated pathways, as well as their association with cytokines in relation to cardiovascular diseases and insulin resistance [25, 27, 28].

Table 5 gives a summary on some of the identified pathways and associations between miRNAs and cytokines in relation to cardiovascular diseases and insulin resistance.

The creation of novel databases comprising noncoding genome sequences has facilitated the development of new therapeutic and diagnostic factors of diseases, since it allows comparisons to be made among a wide range of diseased and nondiseased patients leading to pattern discovery. Database repositories such as circNet (http://circnet.mbc.nctu.edu.tw/) provide tissue specific ncRNA expression profiles and different gene regulatory networks that could be employed in the derivation of different experimental conclusions.

## 10. Conclusion

Noncoding RNAs play a role in regulation of cellular functions including development, proliferation, differentiation, and apoptosis. Abnormalities in these ncRNAs would be reflected in the form of molecular symptoms during disease conditions; thereby the analysis of these could be employed in the conclusion of different diagnostics and development of therapeutic approaches. Insulin resistance and cardiovascular diseases, being two major disease conditions causing morbidity and mortality in the world, require more effective cures and preventive measures. The analysis of ncRNAs related to these disease conditions is considered as a promising research area. With the development of databases, high throughput technologies, and relationships among different ncRNAs and disease conditions, diagnostics could be made in patients and the effects of pathological ncRNAs could be nullified using different approaches.

Since this is a comparatively novel research area there exists a vast number of ncRNAs whose functionalities are yet to be discovered. However, it can be speculated that, given the potential of ncRNAs in regulating cellular processes, this research area is able to provide much needed insight to most pathogenic disease conditions.

## Figures and Tables

**Table 1:** ncRNAs causing insulin resistance related disorders.

ncRNA	Disease condition	Effect	Description
miRNAs			

miR-15a	Chronic glucotoxicity	Negative, prognostic/diagnostic	Overexpression increases the biosynthesis of insulin. Upregulated in short term high glucose levels and downregulated in the long term [11].

miR-34a miR-146 miR-21 miR-29 miR-200	*β*-cell apoptosis	Negative, prognostic	Critical factor leading to diseases related to insulin resistance [11].

miR-375 miR-124a	Hyperglycemia	Negative, prognostic/diagnostic	Suppresses target genes for insulin secretion. Affects the *β*-cell mass. Elevated amounts found in patients with type 2 diabetes. Targets the myotrophin mRNA [20].

miR-200a miR-200b miR-200c miR-141 miR-429 miR-34a	Diabetes	Negative, diagnostic	Highly expressed in the pancreatic islets of diabetic mice [20].

miR-150 miR-192 miR-27a miR-320a miR-375	Type 2 diabetes	Negative, diagnostic	Upregulation is seen [20].

lncRNAs			

Metastasis Associated Lung Adenocarcinoma Transcript 1 (MALAT1)	Found to be a candidate for gene regulation in islets	—	Highly conserved and abundantly expressed in pancreas and other cell types, altered regulation is seen with disease conditions [14].

**Table 2:** ncRNAs causing cardiovascular diseases.

ncRNA	Disease condition	Effect	Description
miRNAs			

miR-378 miR-122 miR-29 miR-26	Cardiac fibrosis	Negative, prognostic/diagnostic	Abnormal expression could induce cardiac fibrosis targeting TFG-*β* including miRNA [15]

miR-126 miR-17-92 cluster	Endothelial dysfunction and inflammation	Negative, prognostic	Negative regulation of angiogenesis in epithelial cells [9]

miR-221 miR-222 miR-155	Initial stages of atherosclerosis	Positive, therapeutic	Inflammatory response of endothelial cells and showing antiangiogenic effects [9, 22]

miR-143 miR-145	Injured or atherosclerosis	Negative, prognostic	Regulation of phenotype of VSMCs in response to injury [9]

miR-21 miR-146a	Vascular injury	Positive, therapeutic	Phenotypic modulation of VSMCs [9]

miR-208a	Acute Myocardial Infarction (AMI)	Negative, prognostic/diagnostic	Causes hypertrophic cardiac growth and fibrosis [13]

miR-23a	Pathological hypertrophy	Negative, prognostic/therapeutic	Upregulated as a result of hypertrophic stimuli in cardiomyocytes, downregulation can be done by ASO [13]

miR-29 miR-24 miR-320	Fibrosis and scar formation	Negative, prognostic	Expression levels drop after a myocardial infarction [13]

miR-19 miR-21 miR-146 miR-155 miR-133	Acute Coronary Syndrome (ACS) and coronary artery disease (CAD)	Negative, prognostic/diagnostic	Microparticles from plasma shows elevated amounts in ACS patients when compared to CAD patients [10, 23]

miR-1 miR-133 miR-208a	Arrhythmias	Diagnostic	The presence of these miRNAs can be used as diagnostic features for arrhythmia, fibrosis, and metabolic disorders [24]
miR-21 miR-29	Fibrosis	Diagnostic	
miR-33	Metabolic disorders	Diagnostic	

circRNAs			

Heart Related Circular RNA (HRCR)	Cardiac hypertrophy and heart failure	Positive, prognostic/therapeutic	Protects the heart from pathological conditions caused by miR-223 by acting as its miRNA sponge resulting in increased amounts of ARC expressions. HRCR is therefore a target for drug development [15]

Cdr1as	Myocardial infarction injuries	Negative, prognostic	miR-7 sponge, the target genes of miR-7 are SP1 and PARP (poly-ADP-ribose polymerase) and they cause cell apoptosis; therefore the reduction in miR-7 increases the cell apoptosis causing SP1 and PARP [15]

Circ-Foxo3	Cardiac senescence	Negative, diagnostic	Highly expressed in patients and causes cells to be held up at G1 phase unable to transit to S phase. Represses cell cycle progression and proliferation [15].

Circular Antisense non coding RNA in INK4 locus (cANRIL)	Atherosclerosis risk	Negative, diagnostic	Regulate atherosclerosis [15]

**Table 3:** ncRNAs affecting both cardiovascular diseases and insulin resistance.

ncRNA	Insulin resistance effect	Cardiovascular effect	Description
miR-34a	Hyperglycemia	Impaired angiogenesis	miR-34a dependent gene regulatory system governs SIRT1 levels and eNOS modifications, both of which affect the two related disease conditions [17].

miR-134	Diabetes mellitus	Microvascular structure formation and reduced cell migration	Highly expressed in patients with the two disease conditions [17].

miR-130a	Diabetes mellitus	Endothelial Progenitor Cell (EPCs) regulation	Diabetes mellitus patients show decreased proliferation, colony formation, and migration but increased apoptosis in EPCs [17].

miR-24	High glucose levels	VSMC pathology	miR-24 targets genes causing high glucose induced cell proliferation and migration [17].

miR-504	Diabetes mellitus	VSMC dysfunction	Upregulation causing both conditions in patients [17].

**Table 4:** Comparison of different technologies available for ncRNA analysis.

Method	Throughput	Cost	Transcript abundance	Produce absolute results	Require a priori knowledge	Accuracy of results
RNA-sequencing	High	Low	Low	Yes	No	High

DNA microarrays	High	Low	High	No	Yes	Depends on transcript abundance

Genomic tiling arrays	High	Low	Low	Yes	No	High

Tissue microarrays	Very high	Low	Low	No	Yes	High

Northern blotting	Low	Low	High	No	Yes	Low

**Table 5:** Associations between miRNAs and cytokines in relation to cardiovascular diseases and insulin resistance.

miRNA	Related cytokines	Effect	Description
miR-34a miR-21 miR-146	Proinflammatory cytokines (Interleukin, IL, and Tumor Necrosis Factor, TNF)	Upregulated miRNA levels with the increase in cytokine levels	MIN6 cells induced with proinflammatory cytokines show a significant induction of miR-34a, miR-21, and miR-146 miRNAs and subsequent blockade of these miRNAs prevented cytokine induced reduction in GSIS and protected *β*-cells from cytokine induced cell death [25].

miR-103 miR-143 miR-107	TNF-*α* (affects insulin and glucose metabolism, provokes insulin resistance, and stimulates lipolysis [26])	Downregulated miRNA levels with the increase in cytokine levels	miR-103, miR-143, and miR-107 are induced in the process of adipogenesis, but after treatment of TNF-*α* their levels have shown to be reduced [25, 27].

miR-99a miR-325	IL-6 (plays a controversial role in the development of insulin resistance and affects glucose metabolism [26])	Downregulated miRNA levels with the increase in cytokine levels	miR-99a and miR-325 show a negative correlation with IL-6 concentration in human subcutaneous and omental adipose tissue [27].

miRNA-125a-5p	IL-6 TNF-*α* IL-2	Downregulated cytokine levels with the increase in miRNA-125a-5p	miRNA-125a-5p mediates lipid uptake and decreases the secretion of inflammatory cytokines such as IL-6, TNF-*α*, and IL-2 [28].

miR-221 miR-222	TNF-*α*	Upregulated miRNA levels with the increase in cytokine levels	miR-221 and miR-222 correlate positively with TNF and negatively with adiponectin [29].
